# Local irradiation does not enhance the effect of immunostimulatory AdCD40L gene therapy combined with low dose cyclophosphamide in melanoma patients

**DOI:** 10.18632/oncotarget.19750

**Published:** 2017-07-31

**Authors:** Sandra Irenaeus, Aglaia Schiza, Sara M. Mangsbo, Jessica Wenthe, Emma Eriksson, Johan Krause, Anders Sundin, Håkan Ahlström, Thomas H. Tötterman, Angelica Loskog, Gustav J. Ullenhag

**Affiliations:** ^1^ Department of Immunology, Genetics and Pathology, Science for Life Laboratory, Uppsala University, 751 85, Uppsala, Sweden; ^2^ Department of Oncology, Uppsala University Hospital, 751 85, Uppsala, Sweden; ^3^ Department of Radiology, Uppsala University Hospital, 751 85, Uppsala, Sweden; ^4^ Department of Surgical Sciences, Uppsala University, 751 85, Uppsala, Sweden

**Keywords:** malignant melanoma, AdCD40L, gene therapy, irradiation, immunotherapy

## Abstract

**Background:**

AdCD40L is an immunostimulatory gene therapy under evaluation for advanced melanoma, including ocular melanoma. Herein, we present the final data of a Phase I/IIa trial using AdCD40L alone or in combination with low dose cyclophosphamide +/- radiation therapy.

**Methods:**

AdCD40L is a replication-deficient adenovirus carrying the gene for CD40 ligand (CD40L). Twenty-four patients with advanced melanoma were enrolled and treated with AdCD40L monotherapy, or combined with cyclophosphamide +/- single fraction radiotherapy. The patients were monitored for 10 weeks using immunological and radiological evaluations and thereafter for survival.

**Results:**

AdCD40L treatment was safe and well tolerated both alone and in combination with cyclophosphamide as well as local radiotherapy. Four out of twenty-four patients had >1 year survival. Addition of cyclophosphamide was beneficial but adding radiotherapy did not further extend survival. High initial plasma levels of IL12 and MIP3b correlated to overall survival, whereas IL8 responses post-treatment correlated negatively with survival. Interestingly, antibody reactions to the virus correlated negatively with post IL6 and pre IL1b levels in blood.

**Conclusions:**

AdCD40L was safely administered to patients and effect was improved by cyclophosphamide but not by radiotherapy. Immune activation profile at baseline may predict responders better than shortly after treatment.

## INTRODUCTION

The incidence of malignant melanoma (MM) has steadily increased in the past decades [1]. Early-stage MM is usually cured by surgery whereas patients diagnosed with stage IV MM only have a 17 percent five-year survival [2]. Recent treatment advances with BRAF and MEK inhibitors and the checkpoint blockade antibodies targeting CTLA4, PD1 or PDL1 have led to improved survival. However, BRAF and MEK inhibitors are only indicated in treatment of patients with BRAF V600 mutated tumors and they and all other available treatments are associated with severe side effects. Moreover, although data is limited, pooled analyses and smaller studies have indicated that checkpoint blockade antibodies are less effective in patients with mucosal MM [3] and there is even less evidence of efficacy in patients with uveal MM [4, 5]. Patients that do not respond to checkpoint blockade therapy may have few tumor infiltrating T cells, which is a prerequisite to respond. Hence, defining other immunotherapeutic options that activate and expand tumor-infiltrating T cells such as local immunostimulatory gene therapy may be of interest.

AdCD40L is a replication-deficient adenovirus carrying the gene for CD40 ligand (CD40L) [6]. We have previously shown that AdCD40L therapy can be used safely in patients with MM [6] and that the T cell to T regulatory cell ratio is significantly enhanced post treatment [7]. The first cohort of patients received four intratumoral injections of AdCD40L and the following cohort received four to eight AdCD40L injections combined with low dose cyclophosphamide conditioning. There is no evidence that cyclophosphamide on its own, and especially not in the low dose applied in our protocol, is of clinical benefit for this patient group. However, cyclophosphamide in low dose may act as an immunostimulatory drug suitable to combine with specific immunotherapy [8]. A few patients experienced clinical benefit but best response as evaluated with whole-body magnetic resonance imaging (WB-MRI) according to RECIST 1.1 was stable disease (SD) [6].

A growing body of evidence indicates that radiotherapy can enhance anti-tumor immune responses in several ways. Irradiation may stimulate the function of NK cells through Smac release from mitochondria [9]. Furthermore, studies indicate that antigen presentation is enhanced by increased cross priming stimulated by radiation-induced cytokine IFN-β, by the soluble danger signal HMGB1 released from dying tumor cells and, by enhanced MHC expression [10–12]. In addition, upregulation of NKG2D ligands and Fas expression are other mechanisms that might contribute to a synergistic effect between immunotherapy and radiotherapy [13, 14].

Hence, to further improve response rates of AdCD40L we added radiotherapy to this treatment regimen and we herein demonstrate safety and effect data. To our knowledge, this is the first clinical study combining radiotherapy with immune stimulating gene therapy.

## RESULTS

### Patients’ characteristics

Twenty-four patients were enrolled in a clinical Phase I/IIa study in Uppsala, Sweden. The effect and clinical description of the first fifteen patients receiving AdCD40L monotherapy or AdCD40L combined with low dose cyclophosphamide have been described previously in an interim report [6]. At this point, an additional nine patients were treated with the addition of radiotherapy. The nine patients with stage IV MM in the third and last cohort of the study were included between March 2014 and January 2015 and consisted of four men and five women with the average age of 65 years, ranging from 55 to 74 years of age. All patients were in good performance status (WHO 0-1) at inclusion. Three patients had skin MM, three patients had ocular MM, two patients had mucosal MM (nasal cavity and perianal area, respectively) and one patient had MM with lymph node as the primary site. All patients had at least two radiologically detectable lesions and were refractory to standard treatment. Background data for all twenty-four patients are summarized in Table 1.

**Table 1:** Background information and overall survival for all malignant melanoma patients treated with AdCD40L

Patient no	Sex	Age^a^	WHO^a^	Primary tumor localisation	Injected metastasis	Number of injections	Previous immunological treatment	LD^b^	Overall survival^c^
**Without cyclophosphamide**									
1	F	72	0	Ocular	Liver	4	No	**14.4**	7
2	F	61	1	Unknown (node)	SC	4	Multiferon	ND	11
3	M	79	1	Ocular	Liver	4	No	ND	13
4*	F	68	0	Ocular	Liver	4	IGF1 inhibitor	2.8	145
5	M	77	0	Skin	SC	4	Interferon	3.1	22
6	M	63	0	Skin	SC	4	No	3.3	21
**With cyclophosphamide**									
7	M	23	0	Ocular	Liver	8	No	3.7	43
8	F	67	0	Mucosal (vulva)	Node	4	No	2.6	216+
9	F	52	0	Ocular	Parotid	4	Methotrexate	3.0	32
10	F	62	0	Ocular	SC	4	No	**4.7**	34
11	F	45	1A	Skin	Node	3	Multiferon, Ipi	**11.8**	5
12	M	63	1A	Skin	Liver	4	Interferon, Ipi	3.8	39
13	F	70	0	Ocular	Liver	4	No	**4.6**	28
14	M	51	1A	Skin	SC	8	Ipi	3.5	64
15	M	61	1	Ocular	Liver	4	No	3.8	13
**With cyclophosphamide and irradiation**									
16*	F	70	0	Ocular	Liver	4	AdCD40L	2.9	19
17	F	69	0	Skin	Node	4	Ipi	**5.3**	33
18	F	56	0	Ocular	Liver	8	No	3.4	65
19	F	69	0	Skin	Liver	4	Pembrolizumab	4.0	45
20	M	55	0	Unknown (node)	SC	4	Ipi	3.2	26
21	F	68	1A	Skin	SC	4	Ipi	3.2	21
22	M	74	0	Mucosal (nasal cavity)	Liver	3	No	2.8	17
23	M	55	1	Ocular	Liver	3	No	**14.3**	6
24	M	70	0	Mucosal (perianally)	Adjacent liver	4	No	2.7	38

Abbreviations: F = female; Ipi = ipilimumab; LD = lactate dehydrogenase; M = male; ND = not done; SC = subcutaneous; WHO pre = performance status at enrollment.

^a^At the initiation of treatment

^b^reference interval: 1.9-4.2 ukat/L

^c^Cut-off: 04 Dec 2016, overall survival in weeks (+: still alive)

*= same patient

The level of lactate dehydrogenase (LDH) for each patient at enrollment is presented in Table 1. Six patients in total had elevated LDH levels at inclusion and three of these patients (#1, #11 and #23) had levels two times above the upper normal limit.

### Safety of AdCD40L treatments

The adverse events judged as probably or possibly related to the study medication are listed in Table 2. They were graded according to common terminology criteria for adverse events (CTCAE) version 4.0. Almost all patients experienced grade 1 to 2 adverse events. None of the patients experienced grade 4 side effects.

**Table 2:** Summary of adverse events according to CTCAE with causality judged as “probably” or “possibly” related to the study medication for all patients with advanced malignant melanoma treated with AdCD40L

Patient no	Pre-treatment biopsy	Injection 1	Injection 2	Injection 3	Injection 4
**Without cyclophosphamide**					
1	-	vomiting (2) pain in the back (2)	-	liver enzyme elevation (3)	vomiting (2)confusion (1) liver enzyme elevation (3)
2	-	-	pain injection site (1)	-	shivering (1) pain muscles (1)
3	-	-	-	liver enzyme elevation (3)	liver enzyme elevation (3) fatigue (2)
4	-	pain injection site (1)	-	pain injection site (1) fever (2)	pain injection site (1)fever (1)
5	-	-	-	-	-
6	-	pain injection site (2)	-	-	pain injection site (2)
**With cyclophosphamide**					
7^1^	-	nausea (1) vomiting (1)	-	common cold (1)	common cold (1) fatigue (1)
7^2^	-	fatigue (1)	-	fatigue (1)	-
8	-	pain injection site (2)	fever (2)	-	-
9	-	vomiting (1)	fever (2)	fever (2)	-
10	-	flu like symptoms (2) swelling of cutaneous metastases (1)	-	-	flu like symptoms (1)
11	-	-	fever with chills (1)	fever with chills (1)	-
12	-	pain related to biopsy (3) fatigue (1)	-	fever with chills (2)	pain after biopsy (3) autoimmune skin reaction (2)
13	-	fever (2)	fever (1)	fever (1)	fever (2)
14^1^	-	-	-	-	-
14^2^	-	-	-	-	-
15	-	fever (1)	-	anorexia (1) fever (2)	fever (1)
**With cyclophosphamide and irradiation**					
16	-	nausea (2)vomiting (2)fever (2)	-	-	-
17	-	-	-	-	-
18^1^	-	post-biopsy pain (2)hoarseness (1)	-	-	-
18^2^	-	-	-	-	-
19	vomiting (1)	-	-	-	-
20	-	nausea (2)common cold (1)	urinary tract infection (2)	-	-
21	-	liver enzyme elevation (1) fatigue (2)	liver enzyme elevation (1) fatigue (1)	liver enzyme elevation (1) fatigue (2)	liver enzyme elevation (2) fatigue (3)
22	-	-	-	fever (1) confusion (2)	-
23	-	-	chills (1)	liver enzyme elevation (3) tachycardia (1) fever (2)	-
24	post-biopsy pain (2)	backache (2)muscle pain (2)	fever (1) nausea (2)	fever (grade 1)	-

Adverse events according to common terminology criteria for adverse events (CTCAE) version 4.0. Grade within parentheses.

^1^First treatment cycle

^2^Second treatment cycle

Of all twenty-four patients, three were hospitalized for reasons assessed to be secondary to the AdCD40L treatment. The hospitalization for patient #12 has already been described in the interim report [6]. Patient #22 was hospitalized in the evening on the day of the third intratumoral AdCD40 injection due to fever (grade 1) and confusion (grade 2) which were assessed as side-effects of the treatment. The patient was discharged after 2 days but was re-hospitalized less than a week later due to general deterioration caused by progressive disease (PD) and the last treatment (fourth injection and second cyclophosphamide infusion) was omitted. Patient #23 was hospitalized after the third intratumoral injection due to fever (grade 2), tachycardia (grade 1), vomiting (grade 2) and general clinical deterioration (grade 2). The fever was judged as an adverse event of treatment and the tachycardia as secondary to fever. The vomiting and decline in performance status were however assessed as secondary to rapid PD. The patient was discharged four days later and the treatment was discontinued.

As described in the interim report, a raise in liver enzymes were noted in some patients during treatment [6]. This was also seen in two patients in the third cohort. None of these patients, including patients in cohort I and II, had normal liver function tests at enrollment and all of them had liver metastases.

### Clinical results according to radiological evaluation

Results of the WB-MRI and positron emission tomography (PET) with [^18^F] fluoro-deoxy-glucose (FDG) combined with computed tomography (CT) or fully integrated PET/MRI system (Signa PET/MR, GE Healthcare, Waukesha, WI) evaluations for all twenty-four patients are listed in Table 3. Omitted radiological evaluations in the first two cohorts have previously been described [6]. Three of the patients in the third cohort (patients #21, #22 and #23) did not undergo all radiological assessments due to rapid PD and clinical deterioration.

**Table 3:** Radiological evaluation by MRI and FDG-PET in malignant melanoma patients with advanced disease treated with AdCD40L

MRI evaluation (RECIST 1.1)			PET evaluation in injected metastasis (EORTC criteria)	
Patient no	Week 5	Week 9	Percent change in SUVmax	Metabolic tumor response
**Without cyclophosphamide**				
1	PD	ND	+9%	SMD
2	PD	ND	-17%	PMR
3	SD	PD	-28%	PMR
4	SD	SD	0	SMD
5	SD	SD	0	SMD
6	PD	ND	+22%	SMD
**With cyclophosphamide**				
7*	SD	SD	-22%	PMR
8	SD	SD	-36%	PMR
9	PD	ND	0	SMD
10	SD	SD	+43%	PMD
11	ND	ND	ND	ND
12	PD	PD	-23%	PMR
13	PD	PD	+40%	PMD
14*	SD	PD	-75%	PMR
15	PD	PD	-25%	PMR
**With cyclophosphamide and irradiation**				
16	PD	PD	+78%	PMD
17	SD	PD	+14%	SMD
18*	SD	SD	-31%	PMR
19	SD	SD	+38%	PMD
20	SD	SD	-34%	PMR
21	PD	ND	-16%	PMR
22	ND	ND	ND	ND
23	ND	ND	ND	ND
24^1^	PD	PD	+47%	PMD

Abrreviations: ND = not done; PD = progressive disease; PMD = progressive metabolic disease; PMR = partial metabolic response; SD = stable disease; SMD= stable metabolic disease.

Treatment response assessments by RECIST 1.1. and EORTC criteria. Change in SUV max in injected metastases at week 5 or week 9 post-treatment evaluations compared to pre-treatment. Largest metabolic change presented. Metabolic response defined as ≥15% decrease in SUVmax and metabolic progression as ≥25% increase in SUVmax.

Week: week post-treatment initiation.

*Radiological evaluation with MRI and PET during the first treatment cycle.

^1^Radiological evaluation done with fully integrated PET/MR system

WB-MRI evaluations of the first fifteen patients have been described previously [6]. Four patients in cohort III had SD at the first WB-MRI evaluation and three patients had PD. Three patients who had SD at the first evaluation continued to have SD whereas one patient had PD at the second WB-MRI evaluation. All patients with PD at the first evaluation continued to have PD at the second evaluation. These results were similar to the results of the MRI evaluations in cohort I and II [6].

Radiological evaluation with PET/CT for patients in cohort I and II has been described in detail previously [6]. Metabolic response is defined according to EORTC criteria as ≥15% decrease in SUVmax and metabolic progression as ≥25% increase in SUVmax [15]. Largest change in metabolic tumor response in the injected metastasis measured by maximum standardized uptake value (SUVmax) at baseline and post-treatment evaluation at week 5 and week 9 expressed as percentage change in SUVmax are summarized in Table 3. In cohort III, three patients had a partial metabolic response (PMR) in the injected metastasis and one patient experienced stable metabolic disease (SMD) whereas three patients had a progressive metabolic disease (PMD). None of the patients with PMD in the injected metastasis at the first PET/CT or PET/MRI evaluation at week 5 had PMR in the injected metastasis on the second PET/CT or PET/MRI evaluation at week 9 compared to baseline in any of the three cohorts. None of the patients in cohort III experienced PMR as assessed by PET/CT or PET/MRI when taking into account all metastases. No obvious differences in PET responses in the injected metastasis could be seen between the different cohorts.

Four patients in total underwent re-treatment with additional cycles of intratumoral injections of AdCD40L. Two of these retreatments (patients #7 and #14) have been described previously [6]. One patient was included twice in the study in different cohorts (#4/16) and the WB-MRI evaluation of both treatment cycles for patient #4/16 are presented in Table 3. Patient #18 had PD according to WB-MRI and PET evaluation at week 5 and week 9 after the second treatment cycle. Unfortunately, it was not possible to measure the change in metabolic tumor response in the injected metastasis during the second treatment cycle in patient #18 as the injected metastasis could not be identified with certainty with PET/CT.

### Overall survival

**Figure 1:**
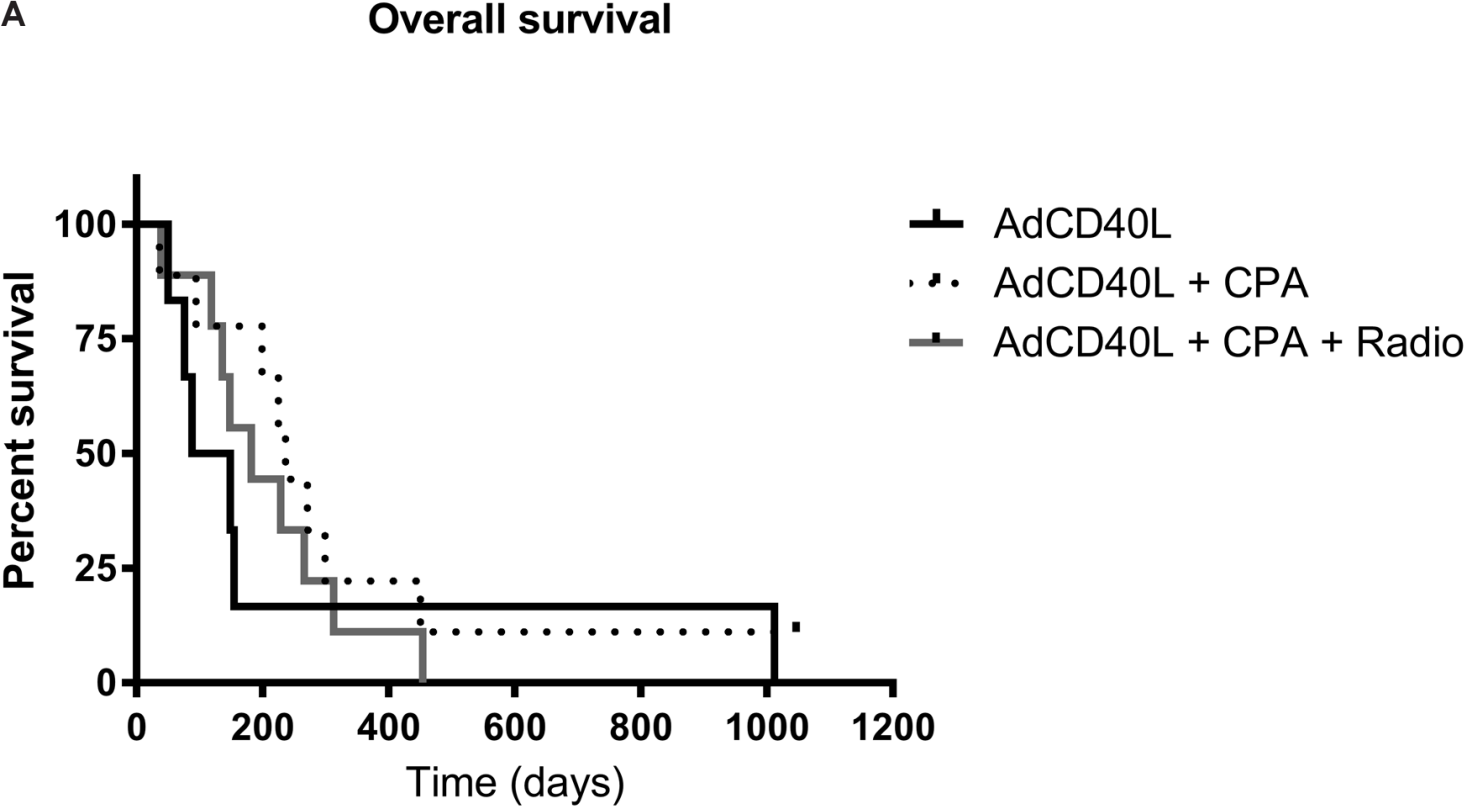

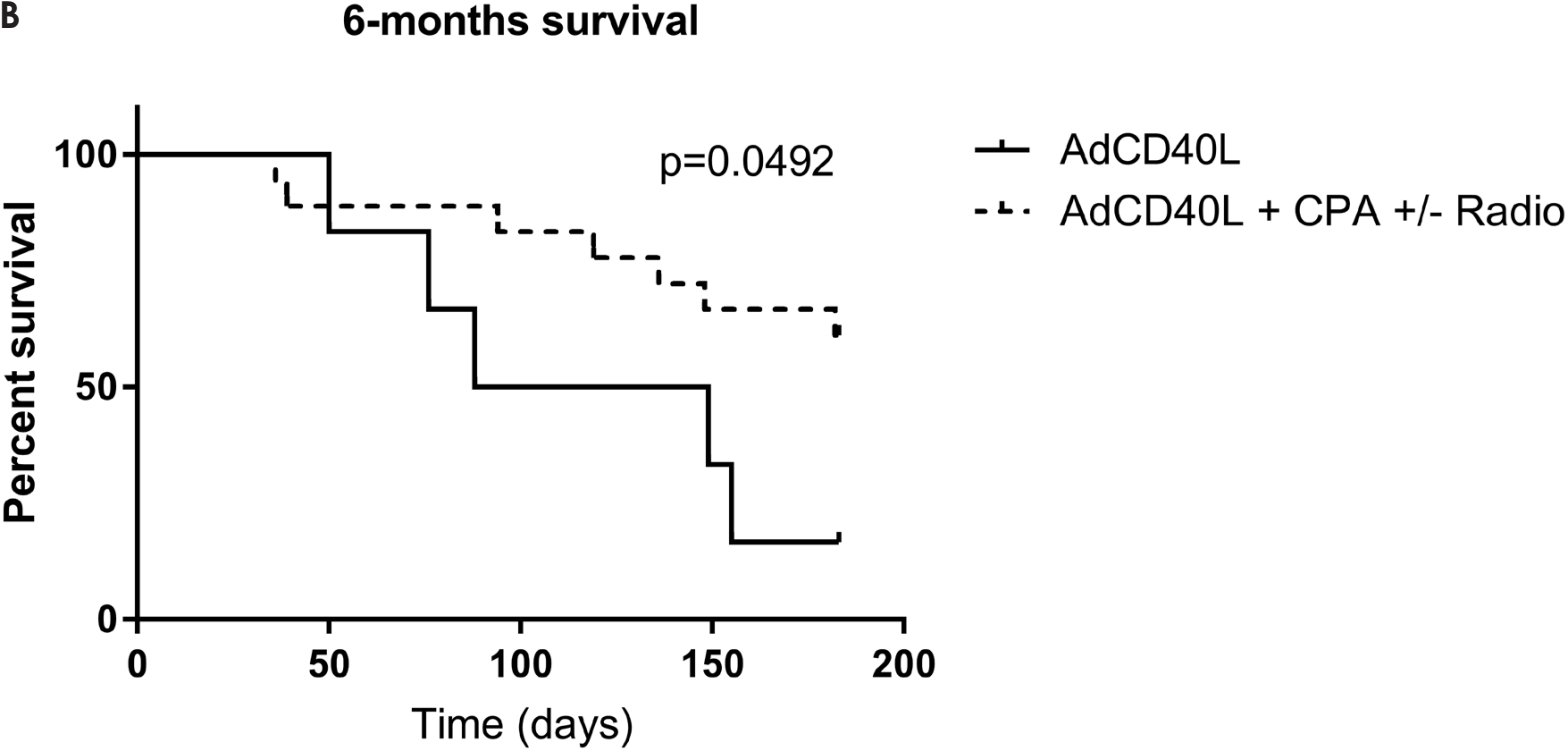
Survival curves for malignant melanoma patients treated with AdCD40L **(A)** Kaplan-Meier survival curves for malignant melanoma patients treated with AdCD40L only (n=6) (black line), AdCD40L combined with low dose cyclophosphamide (n=9) (CPA, dotted line) and AdCD40L combined with cyclophosphamide and 8 Gy single fraction irradiation (n=9) (Radio, grey line). **(B)** Kaplan-Meier survival curves for malignant melanoma patients treated with AdCD40L only (n=6) (black line) and AdCD40L combined with low dose cyclophosphamide +/- 8 Gy single fraction irradiation (n=18) (dotted line). Statistical analysis was done with log rank test.

The median survival in cohort I and cohort II was 17 weeks and 34 weeks respectively [6]. In cohort III the median survival was 26 weeks. Of all twenty-four patients enrolled, thirteen patients had an OS exceeding 6 months and four of these patients had an OS over 1 year (patients #4/16, #8, #14 and #18). At 6 months, 17% of patients were still alive in cohort I, 78% of patients in cohort II [6] and 44% of patients in cohort III. There was no statistically significant difference in OS between patients when comparing all three cohorts (Figure 1A). However, if comparing cohort I in which patients received no conditioning with cohort II and III where conditioning with cyclophosphamide was given, a significantly better 6 month OS was seen (p=0.0492) (Figure 1B). A large proportion of patients in the study had ocular melanoma and the median survival in this subgroup of patients was 28 weeks (6.5 months) and the 1-year OS as well as the 2-year OS 18%.

### Immunological results

**Figure 2:**
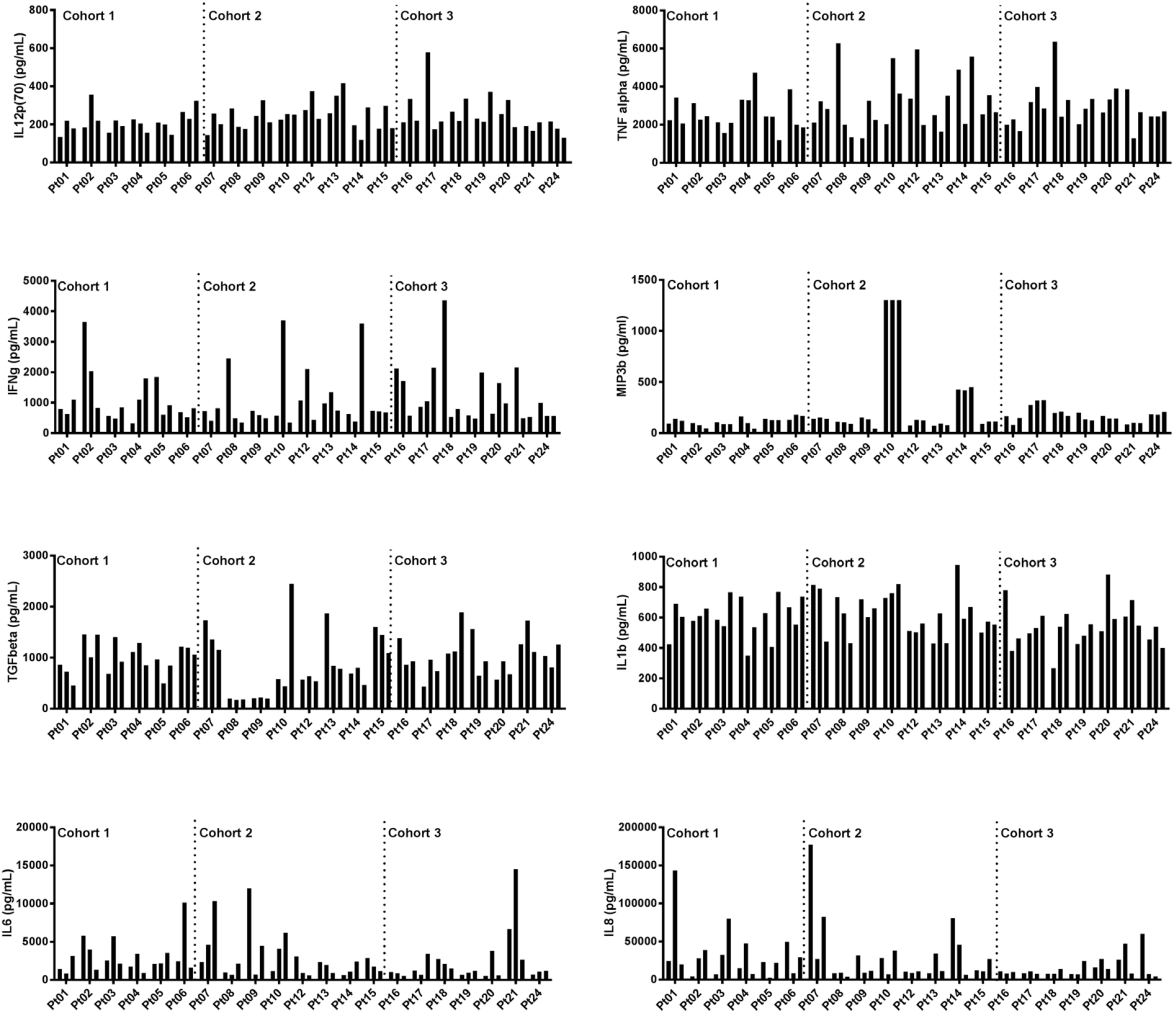
Cohort comparisons of immune marker levels at multiple time points in malignant melanoma patients treated with AdCD40L Plasma concentrations of cytokines detected with multiplex analysis at multiple time points (at baseline, at weeks 3 and 5). Cytokines were divided according to their function as immune stimulators (upper panels) and immune modulators (lower panels). Statistical analysis was done with 2-way Anova/Tukey’s multiple comparison test.

**Figure 3:**
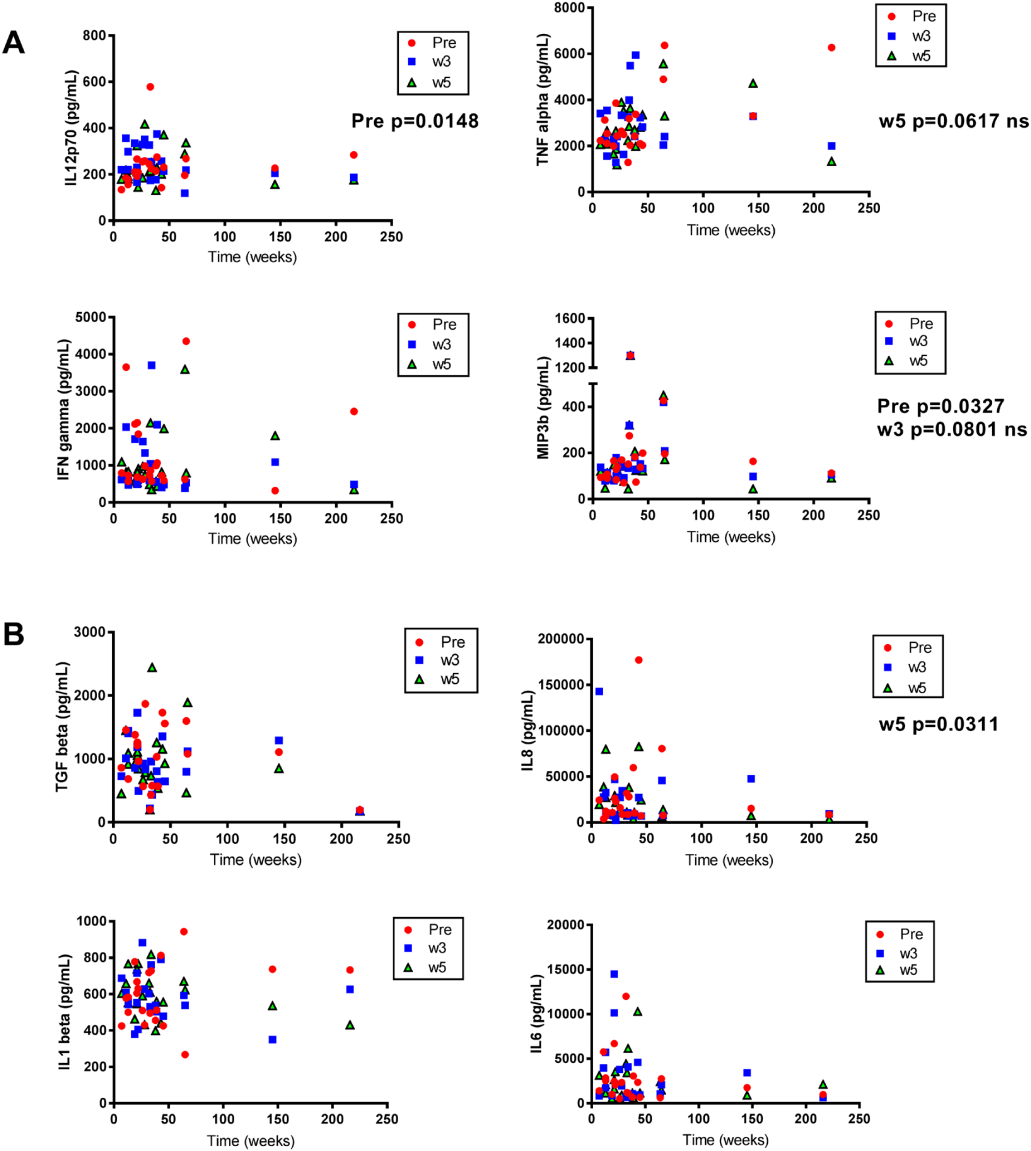
Correlations between immune marker levels and overall survival (OS) in malignant melanoma patients treated with AdCD40L Plasma concentrations of cytokines detected with multiplex analysis at multiple time points (at baseline, at weeks 3 and 5) correlated to OS (weeks). Cytokines were divided according to their function as immune stimulators **(A)** and immune modulators **(B)**. Statistical analysis was done with Spearman’s correlation test.

To investigate immune activation in response to AdCD40L treatments with and without cyclophosphamide and radiotherapy, plasma from different time points was evaluated using Mesoscale multiplex. Plasma from all patients except patient #11, #22 and #23, who clinically deteriorated during the course of the study and therefore did not complete the treatment, was analyzed. There were no significant differences of immune marker levels between the three treatment cohorts (Figure 2). The analytes were divided according to their properties as immune stimulators (Figure 3A) or immune regulators (Figure 3B). The level of IL12 was higher post treatment in most patients but it did not correlate to survival. However, patients with a high initial IL12 concentration had a better OS (Pre, p=0.0148). Initial concentration of MIP3b also correlated with a prolonged survival (Pre, p=0.0327) although the two patients that had a long term survival did not have high MIP3b. If these two individuals are discarded, the level of MIP3b correlates with survival at all time points tested (Pre, p=0.0133; w3, p=0.0034; w5, p=0.0234). IFNγ was increased in individual patients but no statistical significance was shown when correlated to OS.

TGFβ did not correlate to with survival but the patient that is still alive had the lowest TGFβ concentration at any given time point. Similarly, the two long term survivors had low plasma concentration of IL8 and IL6, and decreasing concentrations of IL1b post treatment initiation. Only IL8 concentrations showed a significant negative correlation with survival (w5, p=0.0311).

**Figure 4:**
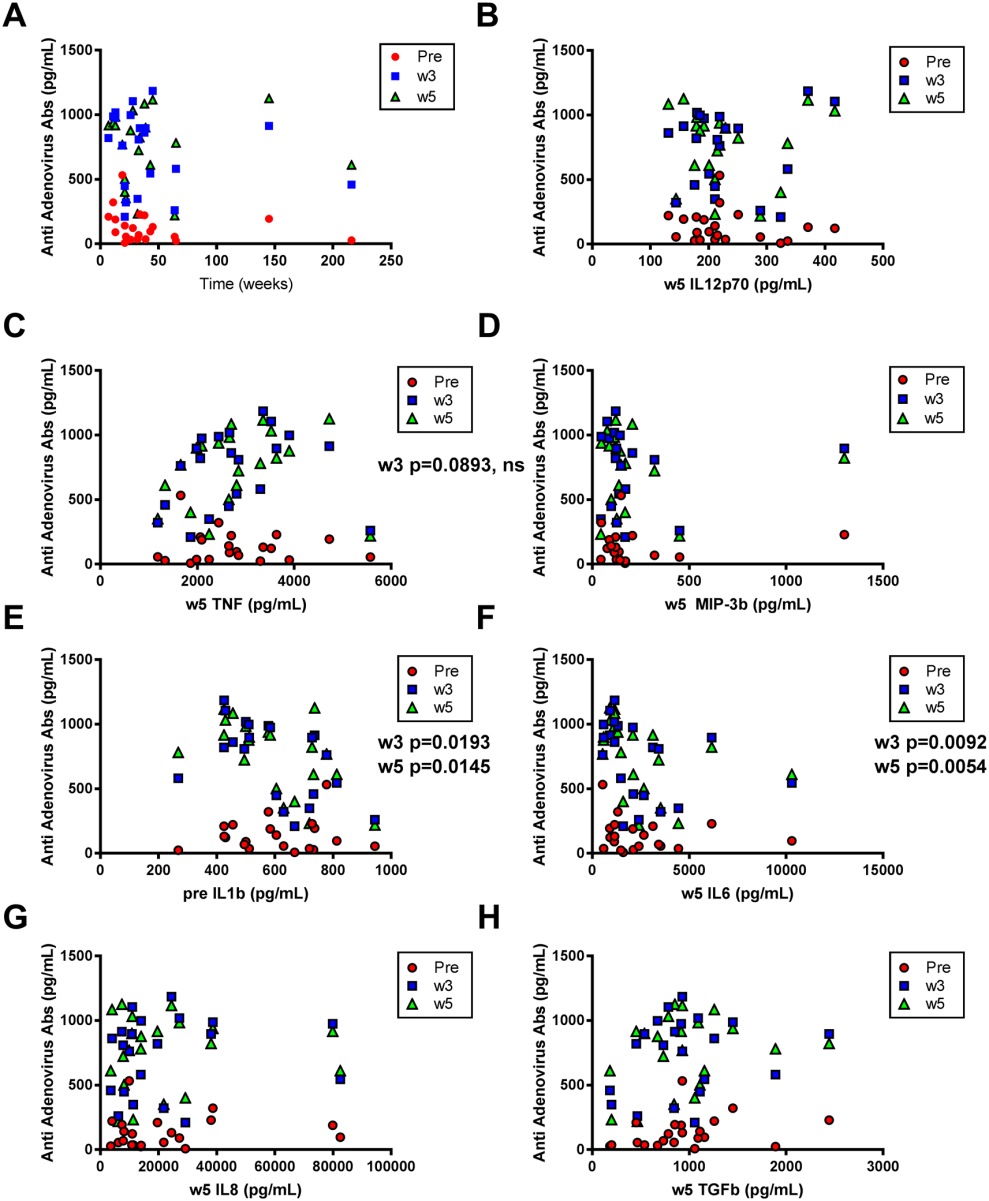
Correlations between anti-adenovirus antibodies and overall survival (OS) and immune marker levels respectively Plasma concentrations of anti-adenovirus antibodies (IgG) detected with multiplex analysis at different time points (at baseline, at weeks 3 and 5) correlated to OS (weeks) **(A)** and to cytokines detected with multiplex analysis at multiple time points (at baseline, weeks 3 and 5) **(B-H)**. Statistical analysis was done with Spearman’s correlation test.

Immune responses directed at the virus were induced post-treatment as seen by the development of anti-adenovirus antibodies. Antibodies targeting adenoviruses were increased in all patients after treatment initiation but the presence of such antibodies did not correlate with OS (Figure 4A). Neither did the anti-adenovirus responses correlate to the level of the immune stimulators IL12, MIP3b or TNF, although the latter tended to correlate to the anti-adenovirus antibodies at week 3 (p=0.0893, ns) (Figure 4B-4D). Interestingly, patients with the lowest level of IL6 post AdCD40L therapy had the highest level of anti-adenovirus antibodies post treatment while a low IL1b level prior treatment correlated to a high antibody level post treatment (Figure 4E-4F). Other time points for each tested molecule did not show significant correlation to antibody levels at any given time point (Figure 4G-4H and data not shown).

## DISCUSSION

Although recent advances in the treatment of metastatic MM have occurred in the past years with the introduction of BRAF and MEK inhibitors as well as anti-CTLA4 and PD1/PDL1 antibodies there is still a great need for new therapies. In the current phase I-IIa study, patients with metastatic MM were treated with intratumoral immunostimulatory gene therapy, AdCD40L. This is the first conducted study with AdCD40L in cancer patients with metastatic disease. We have previously reported results from cohort I (AdCD40L treated only) and cohort II (conditioning with low dose cyclophosphamide) [6]. In this final report, the results from the whole study including cohort III (radiotherapy towards the injected lesion in addition to conditioning with cyclophosphamide) are presented for the first time.

In this phase I/II trial we concluded that it is safe to treat patients with intratumoral injections of AdCD40L, both alone and in combination with low dose cyclophosphamide as well as with local radiotherapy. Almost all patients experienced transient grade 1 and 2 side effects. However, only a few patients experienced grade 3 side effects and none had a grade 4 reaction to treatment. In total, three patients had to be admitted to hospital for reasons related to treatment but could soon thereafter be discharged. Two of these patients had rapid PD and had to discontinue the treatment in advance. No increase in side effects could be seen with advancing age. The frequency of side-effects possibly related to the virus such as fever and flu-like symptoms post-treatment was higher in cohort II compared to cohort I as previously reported [6]. The frequency was not as high in cohort III compared to cohort II but higher than in cohort I. The frequency of fatigue and nausea was slightly higher in cohort II compared to the other cohorts but this could be due to coincidence as the number of patients experiencing these symptoms was low. In conclusion, the radiotherapy did not add toxicity.

We have previously reported that the 6 month OS was significantly better in cohort II as compared to cohort I [6]. When comparing all three groups in a multi-comparison analysis there was no statistically significant difference in OS between the three cohorts. However, patients who received conditioning with cyclophosphamide had a significantly better 6 month OS compared to cohort I. This indicates that preconditioning with cyclophosphamide potentiates the effect of AdCD40L treatment but there seems to be no additional effect of adding local radiotherapy. The response to treatment was independent of age.

Four patients had an OS over 1 year. Two of these patients had ocular MM, one patient had mucosal MM and one patient had skin MM. The four patients were represented in all three cohorts. Three of these patients (#4/16, #14 and #18) were judged to have good clinical effect of the treatment and were therefore accepted for re-treatment with additional cycles. Patient #8 with mucosal MM experienced the longest survival of all patients in the study and has previously been described in detail and was still alive at data cut-off [6]. Elevated lactate dehydrogenase (LDH) levels are associated with decreased survival in metastatic MM [16]. The three patients with the highest levels of LDH were also the patients with the shortest OS. Although these three patients had a good performance status at inclusion they soon deteriorated clinically and the treatment of patient #11 and #23 was discontinued in advance. It is likely that these patients had rapid PD at baseline and that this was reflected by the high LDH levels indicating the poor prognosis.

Extracutaneous MM are much rarer than skin MM. Ocular MM comprise about 3% of all MM and mucosal MM about 1% and patients with these subgroups have in general a worse OS than patients diagnosed with cutaneous MM [17]. In addition, mucosal MMs do only have a mutated BRAF gene in 6% of cases [18] while uveal MMs are not mutated [19]. Hence, the need for better treatments is especially pronounced for these two subgroups. In our study, the largest proportion of patients had ocular melanoma (11/24) and some had mucosal melanoma (3/24) which can be explained by the fact that all patients included were refractory to established treatments which are fewer in these two patient groups. Metastatic ocular MM has a very poor prognosis and the most common site for metastases is the liver [20]. With the presence of liver metastases, median survival is 4-6 months with a 1-year survival of 10-15% whereas the median survival without liver involvement is longer; 19-28 months with a 1-year survival of about 76% [20]. For the ocular MM patients included in our study, the median survival was 6.5 months and the 1-year OS as well as the 2-year OS 18% which is somewhat better than expected considering that all except one (91%) had liver metastases.

The best radiological response to treatment evaluated with WB-MRI was SD in cohort III, similarly to patients in cohort II and III [6]. PET/CT-evaluation showed three patients in cohort III with PMR in the injected metastasis. However, these PMR:s can at least partly have been caused by the irradiation given at pretreatment. None of the patients in cohort III experienced an overall partial or complete metabolic response according to PET/CT or PET/MRI evaluations.

Immune screening of plasma samples from the patients, demonstrated three analytes (IL12, MIP3b and IL8) that correlated statistically to OS. Both IL12 and MIP3b are immune stimulatory agents and showed a positive correlation to OS. IL12 is mainly produced by antigen-presenting cells and its production is enhanced by additional signals such as IFNγ and CD40L-CD40 cell to cell interactions. IL12 has many potential anti-tumoral effects. This cytokine stimulates T-cell differentiation to the Th1 subtype, increases the production of IFNγ, potentiates the cytotoxic effect of NK cells and CD8+ T-cells and, has an anti-angiogenic activity in tumors [21]. The statistically significant relationship between the initial levels of IL12 and MIP3b with better OS indicates that these markers may predict response to AdCD40L treatment. Although the level of IL12 post-treatment did not correlate to survival the level post-treatment was higher compared to the level before treatment in the majority of patients which could indicate a positive effect of AdCD40L treatment. IL8, is a chemokine that plays an important role in the tumor microenvironment by promoting angiogenesis and increasing proliferation and survival of tumor cells [22]. We have previously reported that the fold decrease in IL8 post-treatment compared to baseline correlated to longer OS in patients enrolled in the two first cohorts of the study [6]. When analyzing all 24 patients we also found that the concentration of this immune modulatory agent at week 5 showed a significant negative correlation to survival. No significant differences of immune marker levels were observed between the three different cohorts.

Neither the immune or clinical effects, nor the radiological responses indicate that the irradiation given in cohort III adds benefit. Since previous studies support that one high dose instead of multiple lower doses should be delivered, we choose 8 Gy as a single fraction [23]. However, an existing synergistic effect of AdCD40L treatment and radiotherapy cannot be ruled out with an even higher irradiation dose [24]. Additional studies are needed to define the optimal radiotherapy scheme to combine with immunotherapy and some are ongoing (NCT02710253).

In summary, local immunostimulatory gene therapy with AdCD40L in patients with metastatic MM is well-tolerated both alone, in combination with low dose cyclophosphamide and with the addition of local radiotherapy. The effect of AdCD40L was potentiated by conditioning with cyclophosphamide but local radiotherapy did not add any additional benefit. Immune status prior treatment with high IL12 and MIP3b may predict response to AdCD40L treatment as they correlated to OS.

## MATERIALS AND METHODS

### Trial design

This phase I/IIa study (NCT01455259) was conducted in compliance with our protocol and in accordance with the International Conference of Harmonization – Good Clinical Practice guidelines (ICH-GCP), the principles of the Declaration of Helsinki and accordingly to applicable regulatory guidelines. The protocol was approved by the Regional Ethics Committee and the Medical Products Agency.

The primary objective was to evaluate the feasibility of repeated intratumoral AdCD40L injections alone (cohort I), in combination with low dose cyclophosphamide (cohort II) and of AdCD40L injections in combination with cyclophosphamide after radiotherapy (cohort III) in patients with advanced MM who had received established treatments. Secondary end points were immunological and clinical responses

**Figure 5:**
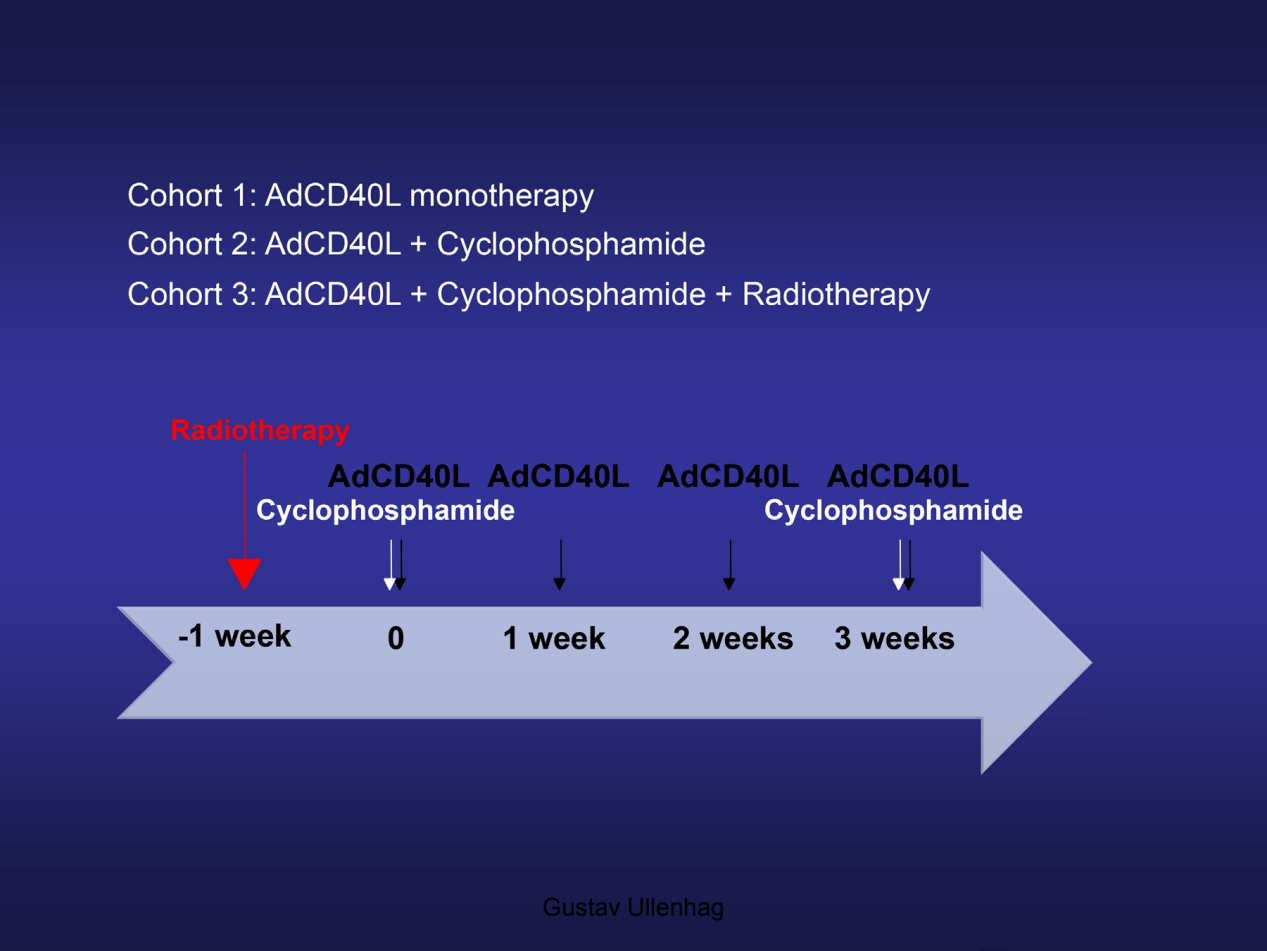
Treatment protocol Treatment protocol for malignant melanoma patients treated with AdCD40L (cohorts I, II and III). Low dose cyclophosphamide at a dose of 300 mg/m^2^ was administered intravenously (cohorts II and III). Radiotherapy of the metastasis selected for AdCD40L injections was delivered as a single 8 Gy fraction (cohort III).

The protocol for the first two cohorts of the study has previously been described in detail [6]. In brief, cohort I of the study included six patients receiving four weekly ultrasound guided intratumoral injections of 2.5 × 10^11^ virus particles AdCD40L. Cohort II included nine patients receiving low-dose cyclophosphamide (300 mg/m^2^) intravenously 1-2 days prior to the first and fourth intratumoral AdCD40L treatment. In cohort III of the study, nine patients were included to receive four intratumoral injections of AdCD40L and conditioning with low dose cyclophosphamide with the addition of an 8 gray (Gy) single-fraction of radiotherapy. The radiotherapy was delivered to the metastasis that had been selected for local treatment one week prior to the first AdCD40L injection. All patients were monitored for 10 weeks during which they were sampled at multiple time points for blood chemistry, hematology and immunology evaluation. [^18^F]fluoro-deoxy-glucose (FDG) positron emission tomography (PET) integrated with CT (FDG-PET/CT) and WB-MRI scans were performed at pretreatment and repeated two (week 5) and six weeks (week 9) after the last AdCD40L injection. The treatment protocol is summarized in Figure 5. The last patient treated did not have to undergo the PET/CT and WB-MRI scans separately since a new machine combining these two modalities had been introduced (fully integrated PET/MRI system, Signa PET/MR, GE Healthcare, Waukesha, WI). Morphological tumor response was evaluated by WB-MRI scan and the MRI part of the PET/MRI according to RECIST 1.1 criteria. Metabolic tumor response was evaluated by FDG-PET/CT and FDG-PET/MRI scans by measuring the maximum standardized uptake value (SUVmax) at one hour after tracer injection where ≥ 15 % decrease in SUVmax was defined as metabolic tumor response and ≥ 25% increase in SUVmax as metabolic progression according to EORTC criteria [15].

Four patients received additional cycles of AdCD40L. One patient was included twice in the study (#4 and #16). This patient was first included in the first cohort of the study and was judged to have had a good clinical effect of the treatment. A little over two years after the first treatment cycle was finished she was therefore enrolled again in the third cohort. Two patients (#7 and #14) were assessed to have had good clinical effect of the treatment and were therefore re-treated with the same protocol as described previously [6]. One patient (#18) did not receive the first and second intratumoral AdCD40L injection in the irradiated metastasis but in a metastasis located in close proximity. This was discovered before the third intratumoral injection was administered and subsequent injections were administrated in the previously irradiated metastasis. The patient remained in good performance status and was judged to have clinical benefit of the given treatment and was therefore re-treated with the same protocol; a previously untreated liver metastasis was irradiated and injected, six months later.

### AdCD40L

AdCD40L is an adenoviral serotype 5, replication deficient vector, which carries the transgene for human CD40L driven by a RSV promoter [25]. The vector was manufactured at Baylor College of Medicine, Houston, Texas. The virus was thawed and diluted to a final dilution of 2.5 × 10^11^ VP in 500 ul Ringer lactate solution and kept at +4 °C before intratumoral injection.

### Analyses of plasma

Patient plasma was analyzed by ELISA for anti-adenovirus antibodies (Adenovirus IgG ELISA, GenWay Biotech Inc, San Diego, CA), TGF-beta1 (Diaclone SAS, Besançon cedex, France) and MIP-3 beta (Nordic BioSite, Täby, Sweden). Meso Scale Diagnostics V-PLEX™ Proinflammatory Panel 1 (MSD, Rockville, MD, USA) was used for detection of IL-12p70, TNF-α, IFN-γ, IL-1beta, IL-6 and IL-8. All assays were performed according to manufacturer’s protocol.

### Statistical evaluations

All statistical analyses were made with Prism Software (Graphpad Software Inc., La Jolla, CA, USA). The difference in 6-months survival between the different groups was evaluated with log-rank test. All correlation analyses were investigated with Spearman’s correlation test and cohort comparison at multiple time points were calculated by 2-way Anova/Tukey’s multiple comparison test. A p-value < 0.05 was considered significant.

## Abbreviations

(MM): malignant melanoma
(PD): progressive disease
(SD): stable disease
(OS): overall survival
(Gy): gray
(LDH): lactate dehydrogenase
(WB-MRI): whole-body magnetic resonance imaging
(PET): positron emission tomography
(18F) (FDG): [^18^F]fluoro-deoxy-glucose
(CT): computed tomography
(PMD): partial metabolic response progressive metabolic disease
(SMD): stable metabolic disease
(SUVmax): maximum standardized uptake value
